# Effect of Tartrazine Dye on Corneoscleral Rim Clarity: An *Ab Externo* Visualization of Cannulated Schlemm’s Canal

**DOI:** 10.3390/jcm15197633

**Published:** 2026-10-02

**Authors:** Lakshminarayanan Gowtham, Sunita Chaurasia, Rashmi Krishnamurthy, Gamal Mohamed, Deepak P. Edward, Khaled K. Abu-Amero

**Affiliations:** 1 Dr. Chigurupati Nageshwara Rao Ocular Pharmacology Research Centre, LV Prasad Eye Institute, Hyderabad 500034, India; gowtham.lakshminarayanan@lvpei.org; 2 Cornea Services, Shantilal Shanghvi Cornea Institute, LV Prasad Eye Institute, Hyderabad 500034, India; sunita@lvpei.org; 3 Glaucoma Services, LV Prasad Eye Institute, Hyderabad 500034, India; rashmikrishnamurthy@lvpei.org; 4 King Faisal Specialist Hospital and Research Center, P.O. Box 3354, Riyadh 11211, Saudi Arabia; olagamal99@gmail.com; 5 Research Department, King Khaled Eye Specialist Hospital and Research Center, Riyadh 11462, Saudi Arabia; dedward@kkesh.med.sa

**Keywords:** tartrazine, corneoscleral rim, refractive index, ab externo, optical clearing, Schlemm’s canal

## Abstract

The intrinsic opacity of the sclera limits *ab externo* visualization of deeper structures such as Schlemm’s canal, thereby constraining angle-based glaucoma surgery. Tartrazine, a US FDA-approved azo dye, transiently enhances tissue optical transparency and may improve intraoperative visualization. This study evaluated whether tartrazine improves the optical clarity of human corneoscleral rim segments (CSRS) and the *ab externo* visibility of Schlemm’s canal cannulation. In this *ex vivo* feasibility (proof-of-concept) study, five human corneoscleral rims from donors unsuitable for corneal transplantation were obtained from Ramayamma International Eye Bank. Two rims were each divided into four quadrants (eight segments in total) and allocated to four groups (*n* = 2 independent segments per group): 0.25 or 0.50 M tartrazine, each without (CSRS-1) and with 6-0 Prolene suture cannulation of Schlemm’s canal (CSRS-2), for 5 min. Segments were examined microscopically at 2.5× magnification, and scleral transparency was compared before and after treatment. Scleral clarity and suture visibility were graded by four independent evaluators on a 0–10 scale, with 10 representing maximum clarity, giving eight scored observations per group from two independent segments. Three further rims (CSRS 5, 6, and 7) underwent histopathological evaluation, comparing tartrazine-treated rims (0.25 and 0.50 M) with untreated rims to assess tissue integrity. Tartrazine at both concentrations increased corneoscleral clarity from an untreated baseline (score 0), with mean clarity scores above 6 in all groups. In non-cannulated segments (CSRS-1), scores were 6.13 at both 0.25 and 0.50 M. In cannulated segments (CSRS-2), suture visibility improved to 6.63 at 0.25 M and 8.00 at 0.50 M, the latter significantly higher than the reference group (*p* = 0.008), although the difference between the two concentrations was not significant (interaction *p* = 0.168). Histopathology showed preserved histological structure with no gross structural damage on light microscopy at either concentration. In this small preliminary series, tartrazine rapidly enhanced scleral translucency and *ab externo* visualization of underlying structures, suggesting potential clinical utility in ophthalmic surgery pending in vivo validation. Larger studies with more independent specimens and objective optical measurements are required to confirm these findings.

## 1. Introduction

Tartrazine (FD&C Yellow No. 5) is a US FDA-approved lemon-yellow synthetic azo dye (21 CFR §74.705). It is a widely used coloring additive in the food industry, pharmaceutical formulations, and cosmetic formulations, and as an adsorption-elution indicator for chloride measurement in biochemistry [1]. Tartrazine was recently shown to induce reversible optical transparency in skin, muscle, and connective tissues of live animals by modulating tissue light scattering [2]. Ou and colleagues (2024) [2] demonstrated that tartrazine can transiently render the opaque abdominal wall of a living mouse translucent. This so-called “transparent abdomen” enabled direct, real-time visualization of fluorescently labeled enteric neurons and gut motility in vivo, without the need for surgical tissue removal or implantation of optical windows [2]. The sclera, which constitutes approximately 83% of the outer fibrous coat of the eye, is opaque due to the irregular diameter and disordered spatial arrangement of its collagen fibrils, which produce intense multiple scattering of light across the visible spectrum [3,4,5]. This structural opacity limits non-invasive visualization of sub-scleral and intrascleral structures and has practical consequences in several areas of clinical ophthalmology. In glaucoma surgery, for instance, identification of Schlemm’s canal (SC), the trabecular meshwork, and the scleral spur is central to the success of *ab externo* procedures such as canaloplasty, viscocanalostomy, and suture-based canal distension. However, scleral opaqueness prevents direct visualization and necessitates reliance on anatomical landmarks, tactile feedback, or intraoperative imaging, which may have variable reliability [6]. Transscleral laser delivery to the ciliary body in refractory glaucoma similarly requires empirical energy titration with no direct anatomical visualization, with recognized risks of both under-treatment and collateral damage [7].

Beyond surgical applications, the sclera constitutes a rate-limiting barrier to transscleral drug delivery: while it is more permeable than the cornea to large hydrophilic macromolecules, its collagen matrix impedes diffusion in a molecular-weight- and charge-dependent manner, and any agent that transiently modifies its optical and structural properties may carry secondary consequences for permeability [8]. Taken together, these considerations provide a strong rationale for exploring reversible, pharmacologically induced scleral transparency as a clinically useful adjunct. The ability to non-invasively visualize deep-seated tissues has important implications for ophthalmic research, in which anatomical structures such as Schlemm’s canal are obscured by the sclera’s intrinsic opacity [4]. Strongly absorbing molecules like tartrazine, at specific wavelengths and when dissolved in water, exhibit Lorentzian resonances that raise water’s refractive index (RI ≈ 1.33) to values close to those of lipid components (RI ≈ 1.43–1.53), as per the Kramers–Kronig relations [2]. The capability of achieving optical transparency in intact ocular tissues has been demonstrated using hydrogel-based clearing protocols combined with light-sheet fluorescence microscopy, which permitted three-dimensional visualization of Schlemm’s canal, blood and lymphatic vessels, corneal nerves, and endothelial cell networks within the intact eyeball [9]. While that approach is not transferable to the living eye, it shows that the optical barriers of ocular tissue are, in principle, surmountable and motivate the search for agents compatible with *in vivo* application. More recently, formulation strategies combining tartrazine with 4-aminoantipyrine have been shown to maintain equivalent clearing performance in live animals while considerably reducing the hepatorenal toxicity associated with the individual dyes at higher concentrations [10], additionally supporting the translational feasibility of absorbing-dye-based tissue clearing.

We hypothesized that tartrazine exposure at the corneoscleral junction or sclera may enhance the transparency of ocular tissues at the corneoscleral rim and assist in suture or device placement in Schlemm’s canal by improving visibility of angle structures. Accordingly, we investigated the feasibility of using tartrazine to enhance the optical clarity of human corneoscleral rim segments and visualization of the SC region. Additionally, the utility of tartrazine treatment in facilitating *ab externo* visualization of Schlemm’s canal cannulation was evaluated using a 6-0 Prolene suture as a contrast marker.

## 2. Materials and Methods

### 2.1. Microscopic Examination and Clarity Scoring

This study was designed as a small-scale feasibility (proof-of-concept) experiment. Two human corneoscleral rims obtained from donors aged 60–70 years deemed unsuitable for corneal transplantation were procured from the Ramayamma International Eye Bank, LV Prasad Eye Institute, Hyderabad, India. The rims were unsuitable for transplantation on the basis of endothelial cell counts measured by specular microscopy, and the donors had no recorded ocular disease. Each corneoscleral rim was divided into four quadrants (i.e., a total of eight corneoscleral rim segments, CSRS). The segments were allocated to one of four groups (*n* = 2 independent tissue segments per group). Groups 1 and 2 received 0.25 and 0.50 M tartrazine, respectively. Groups 3 and 4 were cannulated with 6-0 Prolene sutures and treated with tartrazine at 0.25 M and 0.50 M, respectively [2]. Groups 1 and 2 are referred to collectively as CSRS-1 (without Prolene suture) and groups 3 and 4 as CSRS-2 (with Prolene suture cannulation) throughout the Results and Discussion. Each treated segment was placed on a clean glass slide, exposed to tartrazine for 5 min, and transferred to the microscope stage for evaluation. Tartrazine was applied topically as 100 μL of solution per segment, covering the entire segment. Microscopic assessment was performed at 2.5× magnification using a standard laboratory microscope (Axio Scope.A1, Carl Zeiss, Oberkochen, Germany) equipped with uniform backlighting to assess scleral transparency, which was documented via a digital camera mounted on the microscope. All pre- and post-treatment photographs were obtained under identical, standardized illumination settings to ensure consistent documentation of translucency.

The outcome measures of the study were the degree of improvement in scleral translucency from baseline (untreated) across all groups, and the improvement in *ab externo* suture visibility placed in Schlemm’s canal following tartrazine treatment.

The degree of improvement in scleral clarity at the corneoscleral region and *ab externo* suture visibility following tartrazine treatment was graded by four independent evaluators on a scale of 0 to 10. Untreated sclera was considered the reference standard, with a score of zero (total opacity), while a score of ten represented maximum clarity. Since tartrazine leaves a visible amber-yellow tint on treated tissue, masking to treatment status was not achievable.

Average clarity scores for each treatment group were summarized as mean ± SD with 95% confidence intervals. Untreated specimens served as the baseline condition for descriptive comparisons and were assigned a clarity score of 0 on the rating scale. Because untreated specimens had no variability (all scores = 0), formal statistical comparisons with baseline were not performed; instead, baseline served as a descriptive benchmark, and treatment effects were judged by whether the 95% confidence intervals of the treated groups excluded the baseline score of 0. Each of the four evaluators scored every segment, so each group yielded eight scored observations (four evaluators × two segments). These are repeated assessments of the same two specimens and are not independent biological replicates. To account for the nested and repeated-measures structure of the data, a mixed-effects model was fitted using the CSRS-1 group treated with 0.25 M tartrazine without Prolene sutures as the reference group. The model estimated differences between treatment groups, interaction effects, corresponding 95% confidence intervals, and *p*-values, which are presented in the final two columns of Table 1. A *p*-value < 0.05 was considered statistically significant. The study was not powered to detect differences between concentrations or to define an optimal concentration, and all between-group comparisons were treated as exploratory. Statistical analyses were performed using Stata version 19 (StataCorp LLC, College Station, TX, USA).

### 2.2. Histological Examination

Three human corneoscleral rims (CSRS 5, 6, and 7) were bisected (*N* = 6) and divided into groups 5, 6, and 7 (*n* = 2 per group). Groups 5 and 6 received 0.25 M and 0.50 M tartrazine for 5 min; group 7 was untreated. Saline rinse was performed twice following tartrazine treatment prior to formalin fixation. Residual yellow coloration remained visible after rinsing, and the formalin fixative took on a yellow tint, indicating that some dye was retained in the tissue. The amount of retained dye was not quantified. Samples underwent histopathological assessment on formalin-fixed, paraffin-embedded tissue stained with hematoxylin and eosin to evaluate tissue integrity after short-term exposure.

The overall experimental setup and workflow are shown in Supplementary Figure S1.

## 3. Results

### 3.1. Tartrazine Improved Ab Externo Clarity of Corneoscleral Tissue and Suture Visualization

**Figure 1:**
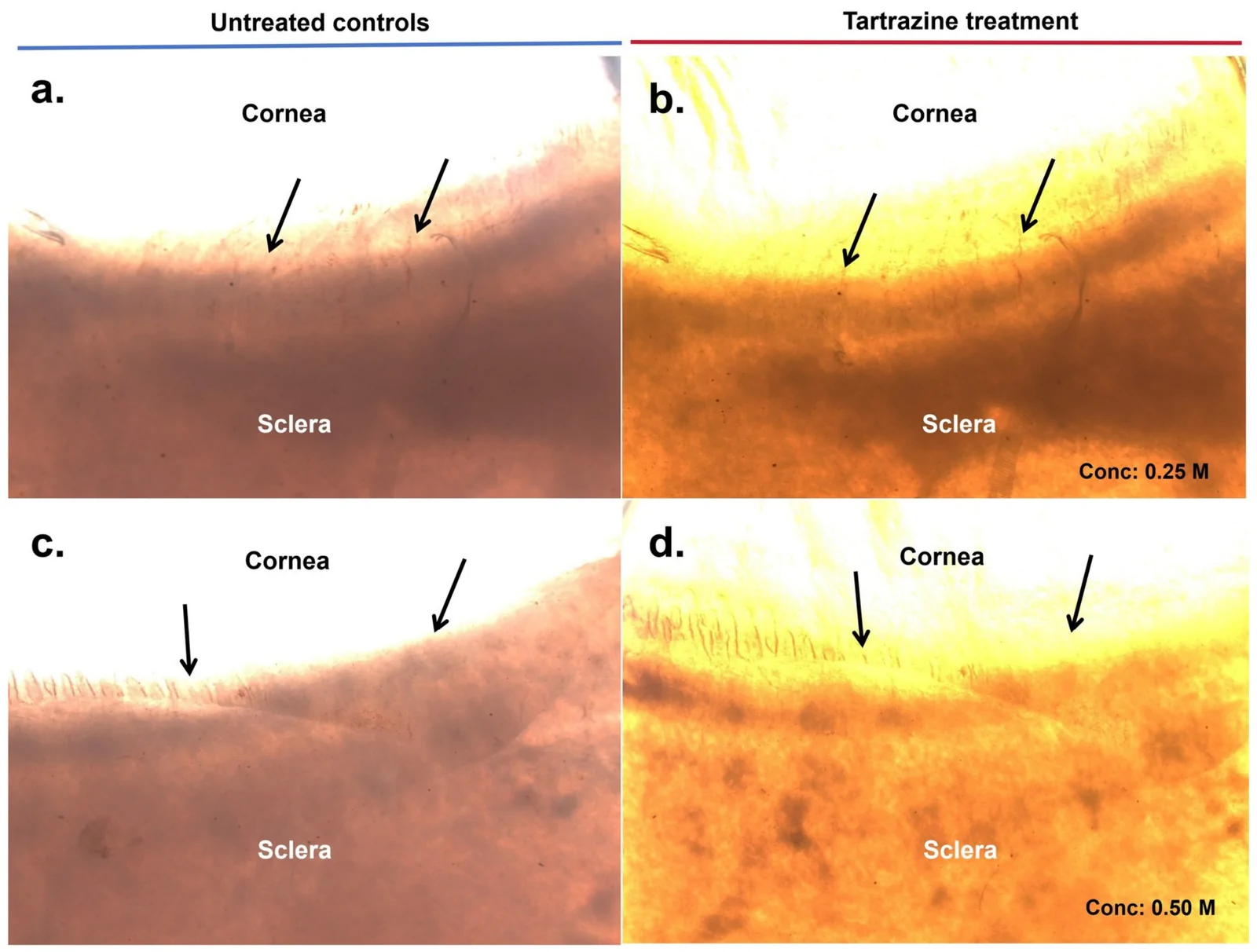
Representative images of the *ab externo* appearance of human corneoscleral rim segments (CSRS) without a Prolene suture, before and after tartrazine exposure. Panels (**a**–**d**) show matched pairs of CSRS-1. In each pair, the left panel (**a**,**c**) shows the untreated baseline, and the right panel shows the same field after treatment. After exposure to 0.25 M (**b**) and 0.50 M (**d**) tartrazine, scleral translucency increased along the corneoscleral segment. The amber-yellow tint in treated panels reflects residual tartrazine dye. Black arrows indicate the corneoscleral junction. All images were taken at 2.5× magnification.

**Figure 2:**
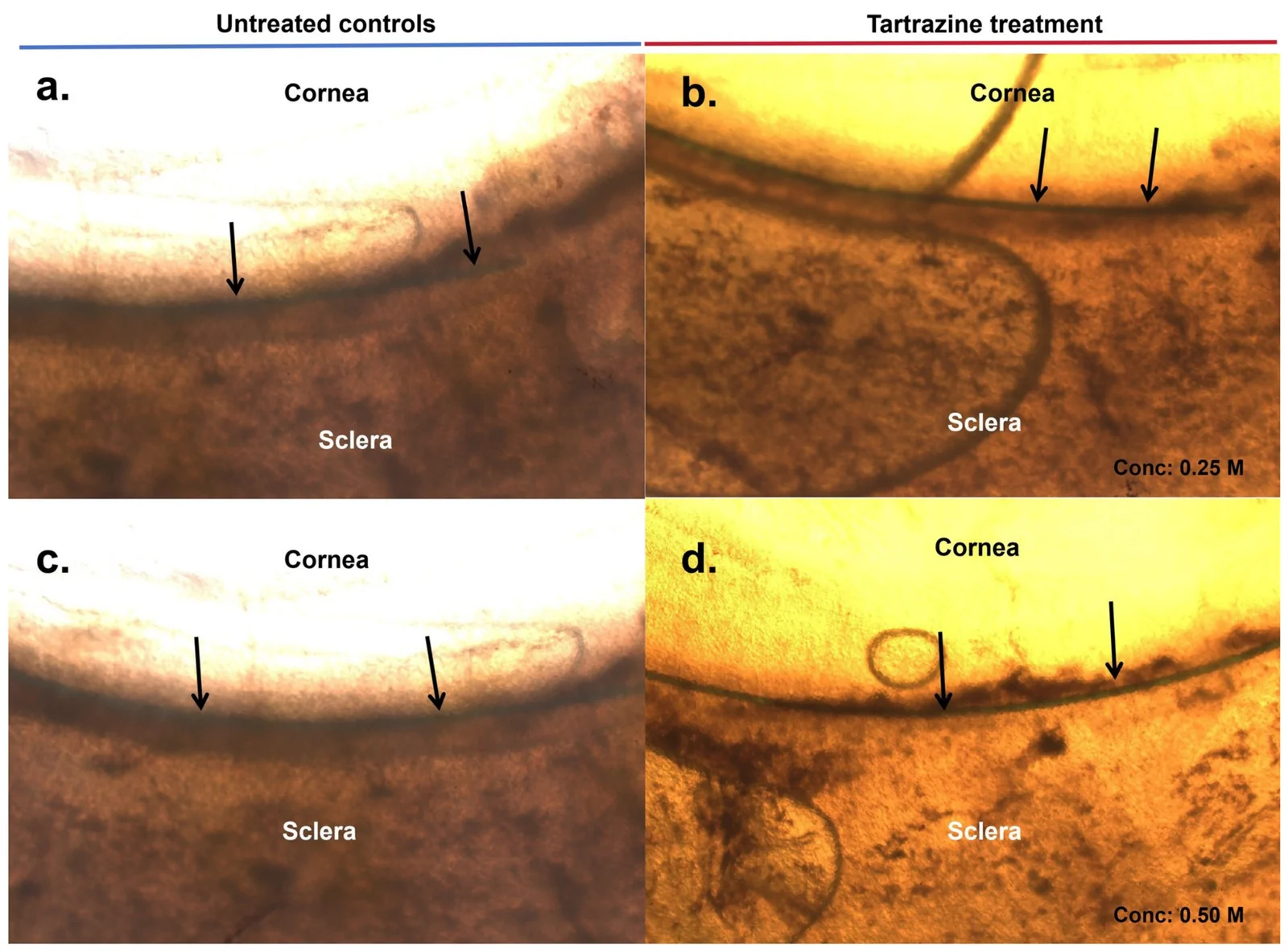
Representative images of the *ab externo* appearance of human corneoscleral rim segments (CSRS) with Prolene sutures, before and after tartrazine exposure. Panels (**a**–**d**) show matched pairs of CSRS-2 (cannulated with a 6-0 Prolene suture through Schlemm’s canal). In each pair, the left panel (**a**,**c**) shows the untreated baseline, and the right panel shows the same field after treatment. After exposure to 0.25 M (**b**) and 0.50 M (**d**) tartrazine, scleral translucency increased along the corneoscleral junction, and the Prolene suture (black arrows) in Schlemm’s canal was more clearly delineated. All images were taken at 2.5× magnification. Note: (**b**,**d**) show air bubble artifacts.

Before tartrazine treatment, CSRS-1 exhibited intrinsic opacity (scored as zero) due to poor light transmittance and limited visualization through the opaque sclera and limbus (Figure 1a,c). Following tartrazine treatment (0.25 and 0.50 M), there was a considerable improvement in light transmittance and translucency through the sclera across all CSRS (Figure 1b,d). In CSRS-2, prior to tartrazine treatment, the Prolene suture was poorly discernible in Schlemm’s canal *ab externo* with background opacity and scleral light scattering (Figure 2a,c). After 5 min of exposure to tartrazine (0.25 and 0.50 M), suture visibility along its track in Schlemm’s canal improved (Figure 2b,d).

### 3.2. Enhanced Scleral Translucency Was Confirmed with Scleral-Clarity Scores Following Tartrazine Exposure

All tartrazine-treated specimens demonstrated substantially higher clarity scores than the untreated baseline condition (score = 0). In every treatment group, the 95% confidence interval for the mean clarity score excluded the baseline value of 0, indicating a clear improvement in scleral clarity following tartrazine exposure. Because each group contained only two independent tissue segments, these results are descriptive and preliminary, and the confidence intervals of the mean, which are based on evaluator-level observations, overstate the precision attainable from two specimens.

**Figure 3:**
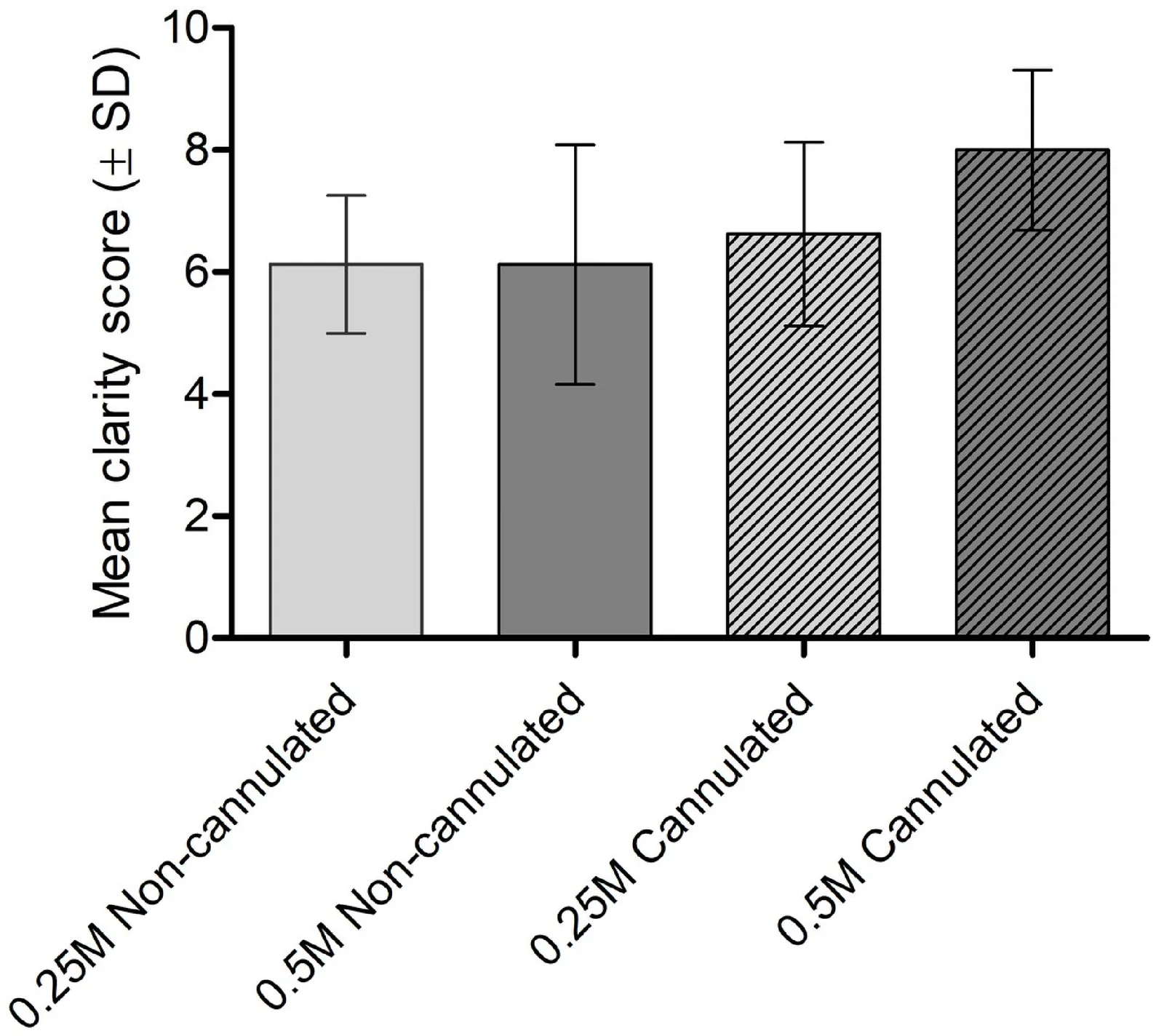
Scleral clarity scores by treatment group. Clarity was assessed on a 0–10 ordinal scale (0 = untreated reference; 10 = maximum clarity). Four independent evaluators each scored four treatment groups: CSRS-1 with 0.25 M tartrazine (light solid bars), CSRS-1 with 0.50 M tartrazine (dark solid bars), CSRS-2 with 0.25 M tartrazine plus Prolene suture (light hatched bars), and CSRS-2 with 0.50 M tartrazine plus Prolene suture (dark hatched bars). Bars represent mean scores; error bars indicate standard deviation. All treatment groups achieved mean clarity scores above 6.0; CSRS-2 at 0.50 M consistently received the highest scores across all evaluators.

Results from the mixed-effects model are expressed relative to the statistical reference group (CSRS-1 treated with 0.25 M tartrazine without Prolene sutures). In CSRS-1 (without Prolene suture), tartrazine exposure at both concentrations increased scleral transparency relative to the untreated baseline (assigned a reference score of 0), yielding mean ± SD clarity scores of 6.13 ± 1.13 and 6.13 ± 1.95 at 0.25 M and 0.50 M, respectively (Figure 3). No statistically significant difference was observed between the two concentrations (mean difference: 0.00, 95% CI: −1.38 to 1.38; *p* = 1.000) (Table 1). The estimates in the final two columns of Table 1 represent comparisons with the model reference category (0.25 M tartrazine without Prolene suture), not comparisons with the untreated baseline.

In CSRS-2 (with Prolene suture cannulation), tartrazine exposure improved *ab externo* suture visibility, with mean ± SD clarity scores of 6.63 ± 1.50 at 0.25 M and 8.00 ± 1.31 at 0.50 M. Exposure at 0.50 M produced a statistically significant improvement in suture visibility compared with the reference group (mean difference: 1.88, 95% CI: 0.49–3.26; *p* = 0.008), whereas the improvement at 0.25 M did not reach statistical significance (mean difference: 0.50, 95% CI: −0.88 to 1.88; *p* = 0.478). Although suture visibility was numerically greater at 0.50 M, no statistically significant difference between the two concentrations was observed (interaction estimate: 1.38, 95% CI: −0.58 to 3.33; *p* = 0.168) (Table 1). Because the model reference is a scleral-clarity score, the estimates for the with-Prolene-suture segments should not be read as within-scale improvements in suture visibility relative to baseline; the within-scale comparison of 0.25 M versus 0.50 M for suture visibility is given by the interaction term (1.38, 95% CI: −0.58 to 3.33; *p* = 0.168).

### 3.3. Histological Assessment Showed Intact Corneoscleral Architecture

**Figure 4:**
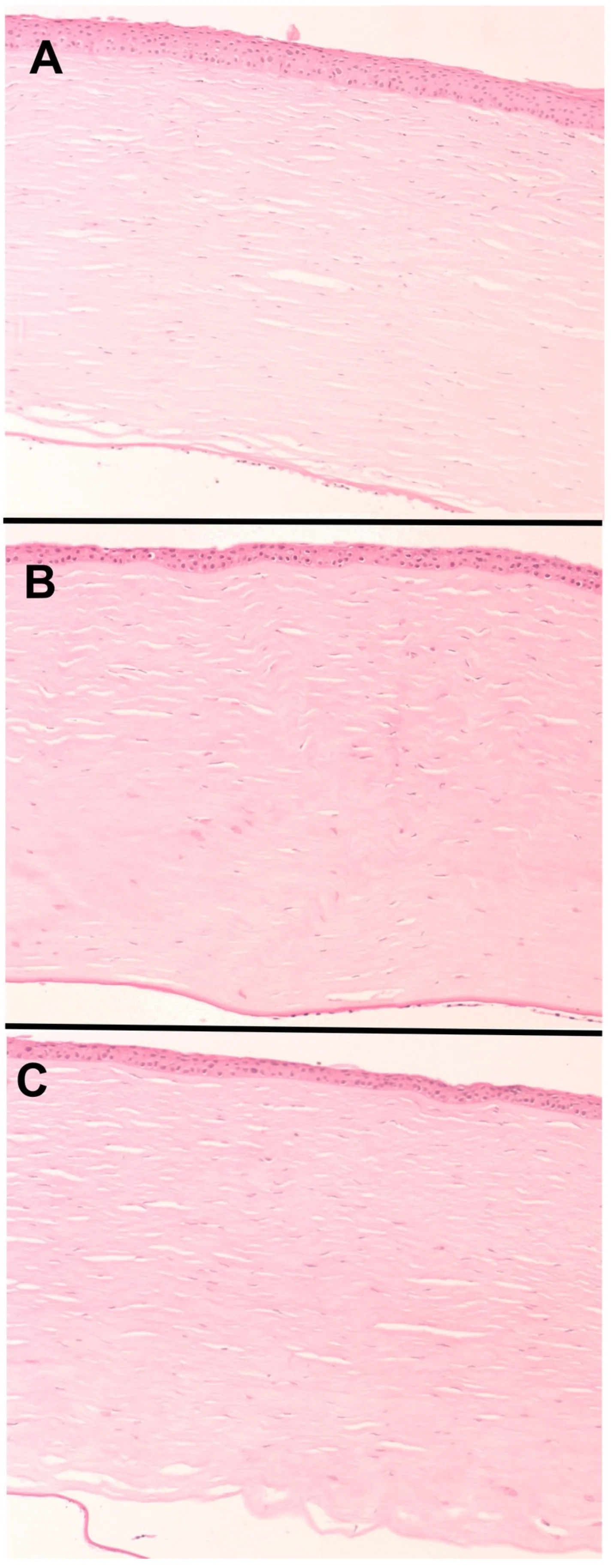
Microphotographs showing assessment of the corneal portion of the corneoscleral rim. (**A**) Untreated control showing normal epithelium, Bowman’s membrane, and underlying stromal architecture. (**B**) 0.25 M at 5 min. (**C**) 0.50 M at 5 min. All groups show intact corneal epithelium and stromal collagen lamellae with regular arrangement, with no evidence of cellular disruption or structural damage, confirming that short-term exposure at both tested concentrations does not compromise tissue morphology (hematoxylin and eosin, 10×).

**Figure 5:**
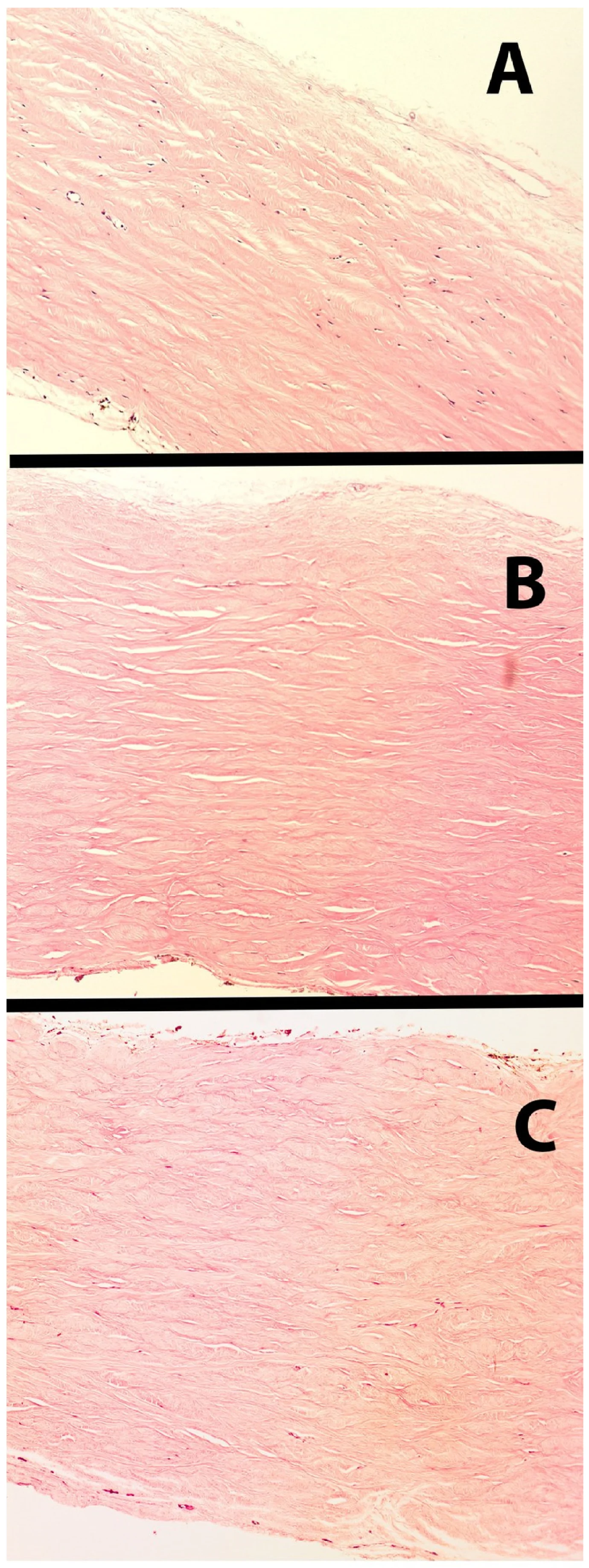
Microphotographs showing the scleral part of the corneoscleral rim in the control and treated groups. (**A**) Control: note the normal scleral collagen architecture and preserved cellular elements. (**B**) 0.25 M tartrazine exposure showing preservation of all structures. (**C**) Exposure to 0.50 M tartrazine, also showing preserved scleral architecture and cellular elements (hematoxylin and eosin, 10× magnification).

Histopathological examination of corneoscleral rim sections (*n* = 6; *n* = 2 per group across groups 5–7; donor age 60–70 years) exposed to tartrazine revealed preservation of overall tissue morphology, comparable to that of untreated controls (Figure 4 and Figure 5). Hematoxylin and eosin-stained sections from corneoscleral rims treated with 0.25 M and 0.50 M tartrazine for 5 min showed intact corneal epithelium, orderly stromal collagen organization in the corneal part of the rim (Figure 4), and no evidence of gross structural disruption in the cornea, limbal region, or sclera (Figure 5).

## 4. Discussion

This study tested the hypothesis that tartrazine, a strongly absorbing azo dye, can enhance the optical clarity of human corneoscleral rim segments and thereby improve *ab externo* visualization of sub-scleral structures, including Schlemm’s canal. The findings support this hypothesis within the limits of a small feasibility study. In CSRS-1 (without Prolene suture), a 5-min tartrazine exposure at both 0.25 M and 0.50 M increased scleral clarity from a baseline of zero to a mean score of 6.13, with no significant difference between concentrations (*p* = 1.000), indicating that no difference between concentrations was detected in corneoscleral tissue without suture placement. However, the small sample size (*n* = 2 independent segments per group) precludes any firm conclusion about the dose–response relationship. In CSRS-2 (with Prolene suture cannulation), tartrazine improved *ab externo* suture visibility, with 0.50 M producing a statistically significant improvement over the reference group (mean difference: 1.88, 95% CI: 0.49–3.26; *p* = 0.008). These results are consistent with the mechanism by which absorbing molecules raise the refractive index of the extracellular aqueous compartment towards that of collagen lipid fractions (RI ≈ 1.43–1.53), thereby reducing scleral light scattering [2,11,12]. That a 5-min application was sufficient to produce this effect in human corneoscleral tissue is the principal finding of this study, and it is the feature most relevant to intraoperative use.

In the Prolene-cannulated CSRS, tartrazine treatment improved suture visibility in Schlemm’s canal *ab externo*. Although Schlemm’s canal is visible *ab interno* for many canal-based procedures using gonioprisms, accurate intraoperative identification of the suture position within Schlemm’s canal is a key determinant of success in *ab externo* angle-based glaucoma procedures such as canaloplasty and viscocanalostomy. Improving scleral clarity could enable canal visualization and prevent misdirected cannulation attempts that carry a risk of failure or tissue damage [6].

The higher score in the 0.50 M CSRS-2 group needs careful interpretation. This observation from a feasibility experiment is consistent with the Prolene suture itself having no effect on scleral transparency when viewed *ab externo*. The suture within Schlemm’s canal is poorly discernible before treatment because it lies beneath the overlying opaque sclera, not because of any optical property of the suture itself. Tartrazine reduces optical scattering in the sclera. Once the overlying opacity is cleared, the suture becomes discernible as a passive sub-scleral marker. The improved score in CSRS-2 therefore reflects scleral optical clearing, not an independent effect of the suture on tartrazine penetration or tissue transparency. The two subgroups were also scored against different targets. In CSRS-1, evaluators graded diffuse scleral translucency, which has no sharp visual end point. In CSRS-2, they graded the visibility of a discrete monofilament. A linear object with defined edges is easier to detect than a gradual change in background translucency, so the two scores are not on a strictly comparable scale. Polypropylene is a hydrophobic, non-absorbent monofilament and is not expected to bind a hydrophilic anionic dye such as tartrazine, so direct interaction between suture and dye is unlikely. With only two independent segments per group, segment-to-segment variation in scleral thickness and pigmentation may also account for part of the difference, and the present data cannot separate these contributions. Experiments that include cannulated segments without dye, and that measure suture-to-background contrast objectively, are needed to determine whether the suture contributes anything beyond serving as a visual marker.

The emergence of *ab interno*, gonioscopy-guided techniques, most notably gonioscopy-assisted transluminal trabeculotomy (GATT), in which a Prolene suture or microcatheter is passed circumferentially through Schlemm’s canal under direct gonioscopic view, may have diminished the need for external canal localization [13]. However, *ab interno* angle surgery is critically dependent on adequate corneal clarity to visualize the angle. In eyes with corneal edema, scarring, or opacification, such as in primary congenital glaucoma with buphthalmos and Haab striae, or in eyes with scarred corneas, the gonioscopic view is precluded, and an *ab externo* trabeculotomy through Schlemm’s canal is preferred [14]. In these settings, the canal cannot be visualized directly, and a suture or probe deviating from Schlemm’s canal may create a false passage into the suprachoroidal space, with attendant risk of bleeding, cyclodialysis, and surgical failure. Tartrazine-induced scleral clearing that renders the suture track and canal lumen visible *ab externo* could therefore allow confirmation of accurate intracanalicular placement and help avert these complications, offering the greatest benefit in eyes where *ab interno* visualization is not possible. In such eyes, the dye could be applied topically to the exposed sclera for a short period before or during canal cannulation, then irrigated, to confirm the intracanalicular course of the suture or catheter. Although only the 0.50 M cannulated group differed significantly from the reference group, the direct comparison of the two concentrations was not significant, so these data do not identify an optimal concentration. The improved delineation of suture course and canal anatomy observed in this study may also assist in recognizing malpositioning and reducing suture-related complications during Schlemm’s canal cannulation with suture placement [15,16], in addition to other possible benefits of scleral clearing [7,8]. Across all four independent evaluators, the highest scores in any group were recorded in CSRS-2 at 0.50 M (Figure 3), with a mean of 8.00 ± 1.31 compared with 6.63 ± 1.50 at 0.25 M. This between-concentration difference did not reach statistical significance (interaction *p* = 0.168); a concentration-dependent enhancement of suture visibility in cannulated tissue therefore cannot be confirmed and would require a larger sample to establish. Because the model reference category is a scleral-clarity score rather than a suture-visibility score, the between-group estimates for the cannulated segments should not be interpreted as within-scale gains in suture visibility relative to an untreated baseline; the within-scale comparison of the two concentrations is given by the interaction term. The resulting improvements in tissue transparency, suture visualization, and Schlemm’s canal trajectory identification could enhance operative precision in anterior segment procedures, but this remains to be shown in vivo.

These findings are consistent with those obtained in ocular tissue by Narawane and colleagues [17], who applied tartrazine to ex vivo porcine and human sclera and demonstrated a progressive, time-dependent increase in optical coherence tomography (OCT) signal penetration through the full scleral thickness, ultimately achieving near-complete visualization of the underlying choroid, including individual choroidal vessels that were wholly obscured in matched controls, together with resolution of suprachoroidal injectate. That study used a 0.4 M tartrazine solution applied by immersion for up to 120 min. In contrast, the present study produced visible *ab externo* clarity at 0.25 M and 0.50 M, concentrations that bracket this value, after only 5 min of exposure. The convergence of an effective clearing concentration in the 0.25–0.50 M range across two independent tissue models and imaging modalities, both near-infrared OCT of the posterior sclera and visible-light microscopy of the corneoscleral rim, supports the activity of tartrazine as a scleral optical clearing agent. The markedly shorter exposure required in this study is advantageous for intraoperative use. Because Narawane and colleagues observed a progressive increase in clearing with longer immersion, a longer exposure, or a lower concentration applied for longer, may produce different clarity in corneoscleral tissue. Neither condition was tested here.

Histopathological examination on light microscopy showed that short-term tartrazine exposure at both tested concentrations did not disrupt corneoscleral rim stromal architecture or produce epithelial defects (Figure 4 and Figure 5), consistent with safety profiles reported in non-ocular tissues [2,10]. Thorough saline irrigation after exposure is recommended to prevent post-treatment irritation caused by residual dye. From a regulatory standpoint, tartrazine is one of the most extensively evaluated synthetic colorants, with an acceptable daily intake of 7.5 mg/kg/day and current approval from the US FDA, the European Food Safety Authority, and the Food Safety and Standards Authority of India [18,19,20].

Several limitations should be acknowledged. First, this was a small-scale feasibility (proof-of-concept) study with two independent tissue segments per group. The four evaluator scores for each segment are repeated assessments of the same specimen, not independent biological replicates, so statistical power, precision, and generalizability are limited. The findings should be regarded as preliminary and hypothesis-generating, not as evidence of an optimal concentration or of a concentration-response relationship. Second, the clarity score is a semi-quantitative measure based on visual assessment. It was graded on an ordinal scale without quantitative optical measurements such as spectrophotometric transmittance or refractive index mapping, and masking to treatment status was not achievable because tartrazine leaves a visible amber-yellow tint, so residual inter-evaluator variability, though partially mitigated by the use of four independent assessors, may have influenced scores. Third, the ex vivo study design using donor tissue deemed unsuitable for transplantation may not completely replicate the optical and biological properties of the living human eye. The tissue came from donors without known ocular disease, whereas the eyes most likely to benefit, such as buphthalmic eyes in primary congenital glaucoma, have thinner and structurally altered sclera in which clearing may differ. Fourth, the reversibility kinetics, optimal exposure time and irrigation protocol, safety of single, repeated or prolonged application to the ocular surface, and confirmation of the in vivo safety profile in animal models remain to be characterized in future studies. Residual dye may also temporarily alter tissue color and could interfere with recognition of surgical landmarks or with intraoperative photography. Larger studies with more independent specimens and objective optical endpoints, such as spectrophotometric transmittance, OCT-based signal penetration, or refractive-index measurements, are required to confirm preliminary observations before clinical translation.

## 5. Conclusions

In this small ex vivo feasibility study, a 5-min tartrazine application improved *ab externo* scleral translucency and the visibility of a suture within Schlemm’s canal in human corneoscleral rim segments, with preserved tissue morphology on light microscopy. These preliminary findings suggest potential clinical utility pending in vivo validation, particularly for anterior segment procedures in which *ab interno* visualization is limited. Larger studies with objective optical measurements are required to confirm them.

## Figures and Tables

**Table 1:** Optical clarity scores by treatment group.

CSRS Group	Tartrazine Conc.	Prolene Suture	Mean Optical Clarity Score (SD)	95% Confidence Interval of the Mean †	Estimated Difference vs. Reference Group (95% CI)	*p*-Value **
CSRS-1 (*n* = 8)	0.25 M	Absent	6.13 (1.13)	5.18, 7.07	Reference group	—
CSRS-1 (*n* = 8)	0.50 M	Absent	6.13 (1.95)	4.49, 7.76	0.00 (−1.38, 1.38)	1.000
CSRS-2 (*n* = 8)	0.25 M	Present	6.63 (1.50)	5.37, 7.88	0.50 (−0.88, 1.88)	0.478
CSRS-2 (*n* = 8)	0.50 M	Present	8.00 (1.31)	6.91, 9.09	1.88 (0.49, 3.26)	0.008
Interaction (Tartrazine × Prolene) *	—	—	—	—	1.38 (−0.58, 3.33)	0.168

† Confidence intervals in the 95% confidence interval of the mean column are interpreted relative to the assigned baseline clarity score of 0 (total opacity); this baseline was a reference value assigned before tartrazine treatment, not a separately measured group. * The interaction effect represents the difference-in-differences between the two groups. In CSRS-1 (without Prolene suture), the change between 0.25 M and 0.50 M tartrazine was 0.00, whereas in CSRS-2 (with Prolene suture), the corresponding change was 1.38. ** Confidence intervals and *p*-values were obtained from mixed-model analysis. *n* = 8 denotes the number of scored observations per group (four independent evaluators × two tissue segments). Each group contained only two independent tissue segments. The eight scores are repeated assessments of those two segments and are not independent biological replicates. The 95% confidence intervals (CI) of the mean were calculated from these eight observations and therefore reflect evaluator-level variability.

## Data Availability

The data supporting the findings of this study are available from the corresponding author upon reasonable request.

## Supplementary Materials

The following supporting information can be downloaded at: https://www.mdpi.com/article/10.3390/jcm15197633/s1, Figure S1: Schematic of the experimental setup, showing the human donor corneoscleral rims, division into segments and group allocation, topical application of 100 μL tartrazine per segment for 5 min, the chemical structure of tartrazine, cannulation of Schlemm’s canal with 6-0 Prolene suture, and the saline rinse applied to specimens processed for histology.
